# 2-Amino-3-nitro­pyridinium hydrogen selenate

**DOI:** 10.1107/S1600536809022879

**Published:** 2009-06-20

**Authors:** Samah Akriche, Mohamed Rzaigui

**Affiliations:** a Laboratoire de chimie des Matériaux, Faculté des Sciences de Bizerte, 7021 Zarzouna Bizerte, Tunisia

## Abstract

The asymmetric unit of the title compound, C_5_H_6_N_3_O_2_^+^·HSeO_4_^−^, contains two monoprotonated 2-amino-3-nitro­pyridinium cations and two hydrogen selenate anions which are connected through N—H⋯O and O—H⋯O hydrogen bonds, building chains parallel to the *a* direction. These chains are further connected to each other by weaker C—H⋯O hydrogen-bonding inter­actions, leading to the formation of a three-dimensional network.

## Related literature

For related structures, see: Akriche *et al.* (2009 ▶); Fleck (2006 ▶); Le Fur, Masse & Nicoud (1998 ▶); Nicoud *et al.* (1997 ▶); Maalej *et al.* (2008 ▶).
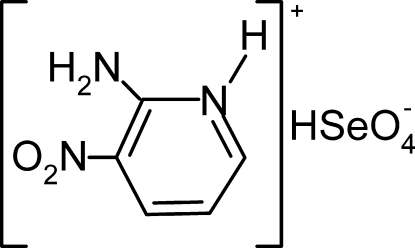


## Experimental

### 

#### Crystal data

C_5_H_6_N_3_O_2_^+^·HSeO_4_^−^
*M* *_r_* = 284.10
Monoclinic, 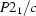

*a* = 9.090 (3) Å
*b* = 20.130 (2) Å
*c* = 10.434 (4) Å
β = 104.84 (2)°
*V* = 1845.6 (10) Å^3^
*Z* = 8
Mo *K*α radiation
μ = 4.09 mm^−1^
*T* = 298 K
0.37 × 0.29 × 0.19 mm

#### Data collection

Enraf–Nonius Turbo-CAD-4 diffractometer
Absorption correction: multi-scan (Blessing, 1995 ▶) *T*_min_ = 0.145, *T*_max_ = 0.298 (expected range = 0.224–0.460)
7325 measured reflections
4433 independent reflections
2980 reflections with *I* > 2σ(*I*)
*R*_int_ = 0.040
2 standard reflections frequency: 120 min intensity decay: 4%

#### Refinement

*R*[*F* ^2^ > 2σ(*F* ^2^)] = 0.047
*wR*(*F* ^2^) = 0.112
*S* = 1.01
4433 reflections
274 parameters
H-atom parameters constrained
Δρ_max_ = 0.80 e Å^−3^
Δρ_min_ = −0.75 e Å^−3^

### 

Data collection: *CAD-4 EXPRESS* (Enraf–Nonius, 1994 ▶); cell refinement: *CAD-4 EXPRESS*; data reduction: *XCAD4* (Harms & Wocadlo, 1995 ▶); program(s) used to solve structure: *SHELXS97* (Sheldrick, 2008 ▶); program(s) used to refine structure: *SHELXL97* (Sheldrick, 2008 ▶); molecular graphics: *ORTEP-3 for Windows* (Farrugia, 1997 ▶) and *DIAMOND* (Brandenburg & Putz, 2005 ▶); software used to prepare material for publication: *WinGX* (Farrugia, 1999 ▶).

## Supplementary Material

Crystal structure: contains datablocks I, global. DOI: 10.1107/S1600536809022879/dn2458sup1.cif

Structure factors: contains datablocks I. DOI: 10.1107/S1600536809022879/dn2458Isup2.hkl

Additional supplementary materials:  crystallographic information; 3D view; checkCIF report

## Figures and Tables

**Table 1:** Hydrogen-bond geometry (Å, °)

*D*—H⋯*A*	*D*—H	H⋯*A*	*D*⋯*A*	*D*—H⋯*A*
O1—H1⋯O6	0.82	1.75	2.565 (5)	170
O5—H5⋯O2^i^	0.82	1.80	2.601 (5)	167
N1—H1*A*⋯O4	0.86	1.84	2.679 (5)	166
N2—H2*A*⋯O3	0.86	2.02	2.870 (6)	172
N2—H2*B*⋯O9	0.86	2.09	2.675 (6)	124
N2—H2*B*⋯O8^ii^	0.86	2.28	2.933 (5)	133
N4—H4⋯O7	0.86	2.05	2.864 (5)	157
N5—H5*A*⋯O8	0.86	2.07	2.900 (5)	163
N5—H5*B*⋯O3^ii^	0.86	2.11	2.798 (5)	137
N5—H5*B*⋯O11	0.86	2.12	2.693 (5)	124
C3—H3⋯O5^iii^	0.93	2.54	3.443 (5)	163
C4—H4*A*⋯O12^iv^	0.93	2.47	3.290 (6)	148
C8—H8⋯O6^iii^	0.93	2.57	3.463 (6)	162
C10—H10⋯O4^v^	0.93	2.35	3.228 (6)	158

Symmetry codes: (i) 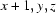; (ii) 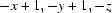; (iii) 
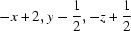
; (iv) 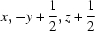; (v) 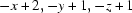.

**Fig. 1.**
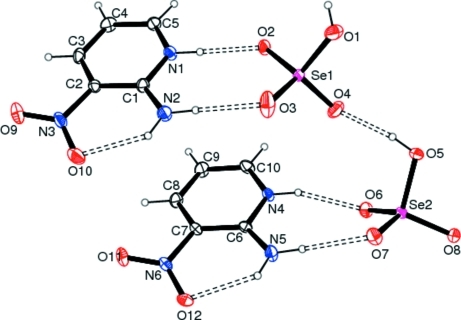
An ORTEP view of (I) with the atom-labelling scheme. Displacement ellipsoids are drawn at the 30% probability level. H atoms are represented as small spheres of arbitrary radii. Hydrogen bonds are represented as dashed lines.

**Fig. 2.**
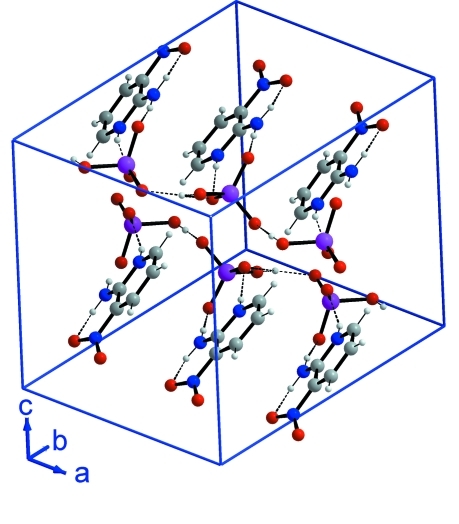
A perspective view of (I) showing the (HSeO4-) n polymeric anions running along the a axis. The C—H···O bonds are omitted for clarity of figure.

## supplementary crystallographic information

### Comment

The important advantage of hybrid organic inorganic salts is the opportunity offered by the chromophores that when anchored onto inorganic host matrices, lead to non-centrosymmetric frameworks suitable for NLO devices. The approach of this new engineering has been applied to 2-amino-3-nitropyridinium cation (2 A3NP^+^) encapsulated in various anionic subnetworks (Akriche *et al.*, 2009, Nicoud *et al.*,1997, Le Fur *et al.*, 1998). The attempt using (HSO_4_^-^) _n_ polymeric anions has been successful with the cristallization of the non-centrosymmetric 2-Amino-3-nitropyridinium sulfate (Le Fur *et al.*, 1998). The encapsulation of this cation in (HSeO_4_^-^)_n_ polymeric anions leads to the title compound (I).

The asymmetric unit of (I) contains two monoprotonated 2-amino-3-nitropyridinium cations and two hydrogen selenate anions (Fig.1) which are connected through N—H···O, C—H···O and O—H···O hydrogen bonds. The hydrogen selenate anions are interconnected between themselves by H-bonds involving the proton of selenate groups leading to (HSeO_4_^-^) _n_ chains parallel to the *a* axis (Fig. 2).

In this atomic arrangement the HSeO_4_^-^ tetraedra are slightly distorted with Se—O distances from 1.594 (6) to 1.713 (4) Å. The Se—O bonds in selenate tetrahedra depends greatly on the nature of the O atoms acting as an acceptor or a donor atoms: the longer bonds (1.713 (4) and 1.692 (5) Å) involve oxygen atoms acting as a H-donor whereas shorter bonds ranging from 1.594 (6) to 1.623 (4) relate to oxygen atoms acting as H-acceptor participating in hydrogen bonds of type N—H···O and C—H···O. As expected, the geometrical features of anion agree with those previously observed for this group in other analogues (Fleck, 2006, Maalej *et al.*, 2008).

The 2-amino-3-nitropyridinium cations are onchored onto anionic chains through short hydrogen bonds originating from the NH_2_ and NH^+^ groups. The unique inter-cation contact C4—H4A···O11(H4A···O11 = 2.45 Å) induces the aggregation of cations in pairs (2 A3NP^+^)_2_ elongated in *-(a+c)* direction. Two hydrogen bonds, N2—H2B···O10 (2.10 Å) and N5—H5B···O12 (2.11 Å) (see Table 1) ensure the intra-cation links. This situation is well observed in nitroaniline derivatives in which nitro and amino groups are *ortho* to one another which precludes the rotation of the nitro group with respect to the pyridinium rings. The diedral angles between the planes of the NO_2_ groups and the two pyridinium planes are 19.0 (3) and 15.9 (4) % indicating a distortion of the NO_2_ groups under the influence of C—H···O hydrogn bonds of neighbouring cations. This situation is alawys observed in other 2-amino-3-nitropyridinium salts (Nicoud *et al.*,1997, Le Fur *et al.*, 1998). The bond lengths and angles in (I) are normal and comparable with the corresponding values observed in the related structure (Akriche *et al.*, 2009, Nicoud *et al.*,1997, Le Fur *et al.*, 1998).

### Experimental

The title compound (I) was cristallized by slow evaporation at room temperature of an aqueous solution (20 ml) of 2-amino-3-nitropyridine (4 mmol) and selenic acid (4 mmol) in a 1:1 stochiometric ratio.

### Refinement

All H atoms attached to C, N and H atoms were fixed geometrically and treated as riding with C—H = 0.93 Å, N—H = 0.86Å and O—H = 0.82 Å with *U*_iso_(H) = 1.2*U*_eq_(C or N) and *U*_iso_(H) = 1.5*U*_eq_(O).

### Crystal data

**Table:**

C_5_H_6_N_3_O_2_^+^·HO_4_Se^−^	*F*(000) = 1120
*M**_r_* = 284.10	*D*_x_ = 2.045 Mg m^−^^3^
Monoclinic, *P*2_1_/*c*	Mo *K*α radiation, λ = 0.71073 Å
Hall symbol: -P 2ybc	Cell parameters from 25 reflections
*a* = 9.090 (3) Å	θ = 9–11°
*b* = 20.130 (2) Å	µ = 4.09 mm^−^^1^
*c* = 10.434 (4) Å	*T* = 298 K
β = 104.84 (2)°	Prism, yellow
*V* = 1845.6 (10) Å^3^	0.37 × 0.29 × 0.19 mm
*Z* = 8	

### Data collection

**Table:**

Enraf–Nonius Turbo-CAD-4 diffractometer	2980 reflections with *I* > 2σ(*I*)
Radiation source: fine-focus sealed tube	*R*_int_ = 0.040
graphite	θ_max_ = 28.0°, θ_min_ = 2.0°
ω scans	*h* = −11→11
Absorption correction: multi-scan (Blessing, 1995)	*k* = 0→26
*T*_min_ = 0.145, *T*_max_ = 0.298	*l* = −6→13
7325 measured reflections	2 standard reflections every 120 min
4433 independent reflections	intensity decay: 4%

### Refinement

**Table:**

Refinement on *F*^2^	Secondary atom site location: difference Fourier map
Least-squares matrix: full	Hydrogen site location: inferred from neighbouring sites
*R*[*F*^2^ > 2σ(*F*^2^)] = 0.047	H-atom parameters constrained
*wR*(*F*^2^) = 0.112	*w* = 1/[σ^2^(*F*_o_^2^) + (0.0549*P*)^2^] where *P* = (*F*_o_^2^ + 2*F*_c_^2^)/3
*S* = 1.01	(Δ/σ)_max_ = 0.001
4433 reflections	Δρ_max_ = 0.80 e Å^−^^3^
274 parameters	Δρ_min_ = −0.75 e Å^−^^3^
0 restraints	Extinction correction: *SHELXL97* (Sheldrick, 2008), Fc^*^=kFc[1+0.001xFc^2^λ^3^/sin(2θ)]^-1/4^
Primary atom site location: structure-invariant direct methods	Extinction coefficient: 0.0106 (7)

### Special details

**Table:**

Geometry. All e.s.d.'s (except the e.s.d. in the dihedral angle between two l.s. planes) are estimated using the full covariance matrix. The cell e.s.d.'s are taken into account individually in the estimation of e.s.d.'s in distances, angles and torsion angles; correlations between e.s.d.'s in cell parameters are only used when they are defined by crystal symmetry. An approximate (isotropic) treatment of cell e.s.d.'s is used for estimating e.s.d.'s involving l.s. planes.
Refinement. Refinement of *F*^2^ against ALL reflections. The weighted *R*-factor *wR* and goodness of fit *S* are based on *F*^2^, conventional *R*-factors *R* are based on *F*, with *F* set to zero for negative *F*^2^. The threshold expression of *F*^2^ > σ(*F*^2^) is used only for calculating *R*-factors(gt) *etc*. and is not relevant to the choice of reflections for refinement. *R*-factors based on *F*^2^ are statistically about twice as large as those based on *F*, and *R*- factors based on ALL data will be even larger.

### Fractional atomic coordinates and isotropic or equivalent isotropic displacement parameters (Å^2^)

**Table:**

	*x*	*y*	*z*	*U*_iso_*/*U*_eq_	
Se1	0.39573 (5)	0.57437 (2)	0.26743 (5)	0.03205 (15)	
Se2	0.88010 (5)	0.61095 (2)	0.28019 (5)	0.02924 (14)	
O1	0.4598 (4)	0.65134 (18)	0.2463 (5)	0.0680 (13)	
H1	0.5519	0.6530	0.2786	0.102*	
O3	0.3344 (5)	0.5426 (2)	0.1228 (4)	0.0756 (14)	
O2	0.2627 (4)	0.58545 (19)	0.3409 (4)	0.0576 (11)	
O4	0.5374 (4)	0.53198 (15)	0.3555 (3)	0.0411 (8)	
O5	1.0304 (3)	0.66505 (15)	0.3064 (4)	0.0401 (8)	
H5	1.1091	0.6443	0.3109	0.060*	
O6	0.7514 (3)	0.65653 (16)	0.3186 (4)	0.0433 (8)	
O7	0.9371 (4)	0.54843 (15)	0.3783 (3)	0.0405 (8)	
O8	0.8397 (4)	0.58792 (17)	0.1281 (3)	0.0478 (9)	
O9	0.4200 (5)	0.2899 (2)	−0.0205 (4)	0.0656 (12)	
O10	0.6241 (5)	0.23103 (19)	0.0156 (4)	0.0672 (12)	
O11	0.8828 (4)	0.33319 (18)	−0.0323 (4)	0.0498 (9)	
O12	1.0837 (4)	0.27232 (16)	−0.0052 (4)	0.0503 (9)	
N1	0.6363 (4)	0.41805 (19)	0.2743 (4)	0.0361 (9)	
H1A	0.6010	0.4565	0.2869	0.043*	
N2	0.4378 (4)	0.4105 (2)	0.0899 (4)	0.0442 (10)	
H2A	0.4098	0.4494	0.1082	0.053*	
H2B	0.3862	0.3897	0.0211	0.053*	
N3	0.5509 (5)	0.2774 (2)	0.0413 (5)	0.0454 (10)	
N4	1.1029 (4)	0.44386 (17)	0.2922 (4)	0.0333 (9)	
H4	1.0723	0.4821	0.3116	0.040*	
N5	0.9152 (4)	0.4497 (2)	0.0988 (4)	0.0413 (10)	
H5A	0.8914	0.4881	0.1236	0.050*	
H5B	0.8653	0.4335	0.0241	0.050*	
N6	1.0111 (5)	0.31667 (18)	0.0290 (4)	0.0348 (9)	
C1	0.5601 (5)	0.3828 (2)	0.1677 (5)	0.0337 (10)	
C2	0.6231 (5)	0.3199 (2)	0.1535 (5)	0.0339 (10)	
C3	0.7524 (5)	0.2982 (2)	0.2424 (5)	0.0400 (12)	
H3	0.7922	0.2568	0.2303	0.048*	
C4	0.8245 (5)	0.3366 (3)	0.3493 (5)	0.0427 (12)	
H4A	0.9114	0.3214	0.4103	0.051*	
C5	0.7647 (5)	0.3970 (2)	0.3629 (5)	0.0381 (11)	
H5C	0.8120	0.4243	0.4333	0.046*	
C6	1.0279 (5)	0.4158 (2)	0.1752 (4)	0.0288 (9)	
C7	1.0836 (5)	0.3528 (2)	0.1496 (4)	0.0286 (9)	
C8	1.2031 (5)	0.3237 (2)	0.2393 (5)	0.0387 (11)	
H8	1.2366	0.2819	0.2216	0.046*	
C9	1.2739 (6)	0.3557 (3)	0.3550 (5)	0.0443 (12)	
H9	1.3562	0.3363	0.4150	0.053*	
C10	1.2217 (5)	0.4159 (2)	0.3799 (5)	0.0402 (11)	
H10	1.2680	0.4382	0.4580	0.048*	

### Atomic displacement parameters (Å^2^)

**Table:**

	*U*^11^	*U*^22^	*U*^33^	*U*^12^	*U*^13^	*U*^23^
Se1	0.0214 (2)	0.0312 (2)	0.0401 (3)	0.00508 (17)	0.00162 (18)	−0.0022 (2)
Se2	0.0249 (2)	0.0232 (2)	0.0358 (3)	−0.00061 (17)	0.00088 (18)	−0.00011 (18)
O1	0.0320 (19)	0.040 (2)	0.129 (4)	0.0059 (17)	0.015 (2)	0.030 (2)
O3	0.079 (3)	0.075 (3)	0.049 (3)	0.045 (2)	−0.026 (2)	−0.021 (2)
O2	0.040 (2)	0.055 (2)	0.088 (3)	0.0112 (18)	0.034 (2)	0.009 (2)
O4	0.0365 (17)	0.0333 (17)	0.045 (2)	0.0110 (14)	−0.0050 (15)	−0.0029 (15)
O5	0.0281 (16)	0.0327 (17)	0.057 (2)	−0.0049 (13)	0.0061 (16)	−0.0020 (16)
O6	0.0299 (17)	0.0338 (17)	0.066 (2)	0.0023 (14)	0.0125 (16)	−0.0058 (17)
O7	0.0416 (18)	0.0311 (16)	0.048 (2)	0.0056 (14)	0.0099 (16)	0.0142 (15)
O8	0.055 (2)	0.0375 (18)	0.041 (2)	0.0050 (17)	−0.0043 (17)	−0.0050 (16)
O9	0.054 (2)	0.061 (3)	0.071 (3)	−0.007 (2)	−0.004 (2)	−0.023 (2)
O10	0.084 (3)	0.046 (2)	0.074 (3)	0.004 (2)	0.026 (2)	−0.023 (2)
O11	0.0348 (18)	0.057 (2)	0.050 (2)	−0.0048 (17)	−0.0036 (17)	−0.0155 (18)
O12	0.066 (2)	0.0294 (18)	0.057 (2)	0.0063 (17)	0.018 (2)	−0.0091 (16)
N1	0.037 (2)	0.031 (2)	0.038 (2)	0.0060 (17)	0.0050 (18)	−0.0043 (18)
N2	0.039 (2)	0.041 (2)	0.046 (3)	0.0080 (19)	0.000 (2)	−0.004 (2)
N3	0.055 (3)	0.029 (2)	0.055 (3)	−0.006 (2)	0.019 (2)	−0.0050 (19)
N4	0.039 (2)	0.0234 (17)	0.032 (2)	0.0039 (16)	−0.0007 (17)	−0.0015 (15)
N5	0.046 (2)	0.036 (2)	0.033 (2)	0.0113 (18)	−0.0072 (19)	−0.0061 (17)
N6	0.043 (2)	0.0291 (19)	0.034 (2)	−0.0076 (17)	0.0117 (19)	−0.0040 (16)
C1	0.033 (2)	0.032 (2)	0.037 (3)	0.0022 (19)	0.010 (2)	0.003 (2)
C2	0.034 (2)	0.031 (2)	0.038 (3)	−0.0001 (19)	0.012 (2)	0.000 (2)
C3	0.042 (3)	0.027 (2)	0.055 (3)	0.008 (2)	0.020 (2)	0.007 (2)
C4	0.037 (3)	0.047 (3)	0.042 (3)	0.009 (2)	0.005 (2)	0.014 (2)
C5	0.035 (2)	0.041 (3)	0.035 (3)	0.002 (2)	0.003 (2)	−0.001 (2)
C6	0.032 (2)	0.029 (2)	0.023 (2)	−0.0036 (18)	0.0041 (19)	0.0005 (18)
C7	0.035 (2)	0.023 (2)	0.029 (2)	−0.0039 (17)	0.0094 (19)	−0.0007 (18)
C8	0.043 (3)	0.027 (2)	0.047 (3)	0.006 (2)	0.012 (2)	0.005 (2)
C9	0.041 (3)	0.045 (3)	0.038 (3)	0.013 (2)	−0.005 (2)	0.005 (2)
C10	0.044 (3)	0.038 (3)	0.031 (3)	−0.003 (2)	−0.005 (2)	0.000 (2)

### Geometric parameters (Å, °)

**Table:**

Se1—O3	1.602 (4)	N4—C10	1.347 (5)
Se1—O2	1.604 (3)	N4—C6	1.359 (5)
Se1—O4	1.620 (3)	N4—H4	0.8600
Se1—O1	1.689 (4)	N5—C6	1.316 (5)
Se2—O8	1.603 (3)	N5—H5A	0.8600
Se2—O6	1.616 (3)	N5—H5B	0.8600
Se2—O7	1.621 (3)	N6—C7	1.456 (5)
Se2—O5	1.713 (3)	C1—C2	1.412 (6)
O1—H1	0.8200	C2—C3	1.369 (7)
O5—H5	0.8200	C3—C4	1.377 (7)
O9—N3	1.225 (5)	C3—H3	0.9300
O10—N3	1.216 (5)	C4—C5	1.355 (7)
O11—N6	1.224 (5)	C4—H4A	0.9300
O12—N6	1.217 (5)	C5—H5C	0.9300
N1—C1	1.352 (6)	C6—C7	1.417 (6)
N1—C5	1.357 (6)	C7—C8	1.370 (6)
N1—H1A	0.8600	C8—C9	1.374 (7)
N2—C1	1.321 (6)	C8—H8	0.9300
N2—H2A	0.8600	C9—C10	1.351 (7)
N2—H2B	0.8600	C9—H9	0.9300
N3—C2	1.463 (6)	C10—H10	0.9300
			
O3—Se1—O2	112.5 (2)	O11—N6—C7	118.4 (4)
O3—Se1—O4	111.03 (18)	N2—C1—N1	117.2 (4)
O2—Se1—O4	112.89 (19)	N2—C1—C2	127.9 (5)
O3—Se1—O1	106.9 (3)	N1—C1—C2	114.9 (4)
O2—Se1—O1	105.2 (2)	C3—C2—C1	121.1 (4)
O4—Se1—O1	107.86 (17)	C3—C2—N3	119.1 (4)
O8—Se2—O6	114.36 (18)	C1—C2—N3	119.8 (4)
O8—Se2—O7	110.84 (17)	C2—C3—C4	121.1 (4)
O6—Se2—O7	114.76 (17)	C2—C3—H3	119.4
O8—Se2—O5	108.25 (19)	C4—C3—H3	119.4
O6—Se2—O5	101.41 (16)	C5—C4—C3	117.9 (4)
O7—Se2—O5	106.24 (17)	C5—C4—H4A	121.0
Se1—O1—H1	109.5	C3—C4—H4A	121.0
Se2—O5—H5	109.5	C4—C5—N1	120.4 (5)
C1—N1—C5	124.5 (4)	C4—C5—H5C	119.8
C1—N1—H1A	117.7	N1—C5—H5C	119.8
C5—N1—H1A	117.7	N5—C6—N4	117.6 (4)
C1—N2—H2A	120.0	N5—C6—C7	127.6 (4)
C1—N2—H2B	120.0	N4—C6—C7	114.8 (4)
H2A—N2—H2B	120.0	C8—C7—C6	120.8 (4)
O10—N3—O9	123.6 (5)	C8—C7—N6	118.7 (4)
O10—N3—C2	117.8 (4)	C6—C7—N6	120.4 (4)
O9—N3—C2	118.6 (4)	C7—C8—C9	120.8 (4)
C10—N4—C6	124.6 (4)	C7—C8—H8	119.6
C10—N4—H4	117.7	C9—C8—H8	119.6
C6—N4—H4	117.7	C10—C9—C8	118.7 (4)
C6—N5—H5A	120.0	C10—C9—H9	120.6
C6—N5—H5B	120.0	C8—C9—H9	120.6
H5A—N5—H5B	120.0	N4—C10—C9	120.3 (4)
O12—N6—O11	124.2 (4)	N4—C10—H10	119.9
O12—N6—C7	117.5 (4)	C9—C10—H10	119.9

### Hydrogen-bond geometry (Å, °)

**Table:**

*D*—H···*A*	*D*—H	H···*A*	*D*···*A*	*D*—H···*A*
O1—H1···O6	0.82	1.75	2.565 (5)	170
O5—H5···O2^i^	0.82	1.80	2.601 (5)	167
N1—H1A···O4	0.86	1.84	2.679 (5)	166
N2—H2A···O3	0.86	2.02	2.870 (6)	172
N2—H2B···O9	0.86	2.09	2.675 (6)	124
N2—H2B···O8^ii^	0.86	2.28	2.933 (5)	133
N4—H4···O7	0.86	2.05	2.864 (5)	157
N5—H5A···O8	0.86	2.07	2.900 (5)	163
N5—H5B···O3^ii^	0.86	2.11	2.798 (5)	137
N5—H5B···O11	0.86	2.12	2.693 (5)	124
C3—H3···O5^iii^	0.93	2.54	3.443 (5)	163
C4—H4A···O12^iv^	0.93	2.47	3.290 (6)	148
C8—H8···O6^iii^	0.93	2.57	3.463 (6)	162
C10—H10···O4^v^	0.93	2.35	3.228 (6)	158

Symmetry codes: (i) *x*+1, *y*, *z*; (ii) −*x*+1, −*y*+1, −*z*; (iii) −*x*+2, *y*−1/2, −*z*+1/2; (iv) *x*, −*y*+1/2, *z*+1/2; (v) −*x*+2, −*y*+1, −*z*+1.
